# Perceptions of Nurse–Surgeon Communication in the Operating Room: A Q-Methodology Study

**DOI:** 10.3390/ijerph22020229

**Published:** 2025-02-06

**Authors:** Jeong Hwa An, Eun Ja Yeun, Sul Hee Lee, Ho Young Kim

**Affiliations:** 1 Department of Nursing, Yeoju Institute of Technology, 338 Sejong-ro, Yeoju-si 12652, Gyeonggi-do, Republic of Korea; jhan0802@gmail.com; 2 Department of Nursing, Konkuk University, 268 Chungwon-Daero, Chungju-si 27478, Chungcheongbuk-do, Republic of Korea; 3 Department of Nursing, Kyungmin University, 545 Seobu-ro, Uijeongbu-si 11618, Gyeonggi-do, Republic of Korea; dltjfgml1123@naver.com; 4 Konkuk University Medical Center, 120-1 Neungdong-ro, Gwangjin-gu, Seoul 05030, Republic of Korea; holang77@kuh.ac.kr

**Keywords:** communication, nurse, operating room, perception, surgeon, Q-methodology

## Abstract

Inadequate communication between nurses and surgeons can lead to patient injuries and increased healthcare costs. This study aimed to identify and understand subjective perceptions of communication between Korean nurses and surgeons in operating rooms (ORs), focusing on their values, beliefs, and attitudes. The Q-methodology was employed in this research, as it integrates the strengths of both quantitative and qualitative approaches. A convenience sample of 46 participants classified 45 Q-statements by using a nine-point forced normal grid. The collected data were analyzed by using by-person factor analysis with the pc-QUANL program. This study revealed four distinct types of nurse–surgeon communication in the OR: professional communication, cynical conflict, passive task-oriented communication, and relationship-oriented endurance. These four types accounted for 58.7% of the total variance, distributed as follows: type 1 (38.7%), type 2 (9.8%), type 3 (5.4%), and type 4 (4.8%). The eigenvalues were 15.8, 4.1, 2.2, and 1.9, respectively. Based on these findings, tailored strategies to enhance nurse–surgeon communication according to each type are essential. Enhancing communication dynamics can lead to more effective interactions, improve patient care and safety, and boost job satisfaction among healthcare professionals. The results have significant implications for healthcare organizations and nursing managers aiming to improve nurses’ communication skills. Additionally, this study provides insights for healthcare organizations in other countries regarding the communication competence traits among Korean health professionals.

## 1. Introduction

Communication involves the exchange of opinions and information among organization members or between an organization and its members. Organizations constantly perform organizational activities through intercommunication and can only exist through mutual interaction with other organizations. Effective communication is the interaction between two or more individuals where the intended message is successfully delivered, accurately received and clearly understood concisely and coherently [1]. Hence, effective communication is important for increasing the efficiency of an organization. Furthermore, effective communication is vital in today’s healthcare environment, and poor communication can lead to conflict among healthcare providers. Communication within surgical teams is a critical component of operative efficiency [2]. Factors influencing optimal communication, including team turnover, role composition, and mutual familiarity, continue to be under investigation in the operating room. The operating room (OR) represents a highly technical work environment that necessitates attention to patient care and safety, in addition to surgery and other invasive procedures, making effective communication among team members even more crucial [3].

### 1.1. Literature Review

Effective interprofessional communication plays a critical role in the OR but faces many challenges at the individual, team, environmental, and organizational levels. A standardized information delivery system for medical staff is especially required in operating rooms (ORs) during operations, as it would allow for the immediate delivery and evaluation of information to ensure patient safety [4]. Effective communication resolves misunderstandings between members and can help manage or coordinate their activities. Effective communication and cooperative relationships between professionals have been found to relate to fewer work errors, improved patient outcomes, increased job satisfaction, and reduced work hours, all of which benefit patients and facilitate even better communication [5]. Healthcare is provided through systematic organization and teamwork in a strictly controlled and restricted environment in the OR. Thus, accurate nurse–surgeon communication is important for providing high-quality healthcare [6]. In a previous study, the frequency of communication in the OR between surgeons and operating nurses accounted for a total of 16.0% of all interactions, while surgeon–resident and surgeon–circulating nurse communication accounted for 13.8% and 12.4%, respectively [3]. Thus, nurse–surgeon communication comprised the largest proportion of communicative interactions in the OR. Nine factors were associated with effective nurse–surgeon communication, including accuracy, comprehensibility, timeliness and usefulness, reliability, consistency, balance, repetition, cultural self-confidence, and openness [7]. Furthermore, nurse–surgeon cooperation appears to be strongly correlated with job autonomy, and nurses who felt highly recognized during nurse–surgeon collaboration show higher job autonomy and organizational commitment. Moreover, individual and organizational commitment and usefulness, reliability, consistency, balance, repetition, cultural self-confidence, and openness explained nearly 60.0% of the variance in this attitude [6].

However, job autonomy is significantly lower in the OR than in intensive care units (ICUs) or general wards. Indeed, patient safety and nurse communication appear to substantially differ by work department, with ORs/recovery rooms having comparatively lower values in these factors than the ICU. The turnover rate was also higher among OR nurses compared with nurses working in other units, and ORs show the highest levels of nurse verbal abuse by surgeons and superiors [8]. Overall, these findings indicate that communication in the OR is very important for both patient safety and hospital organizational culture, including the job satisfaction of major agents such as nurses and surgeons. However, there are currently few studies on communication within medical teams in South Korea. Most previous studies have been conducted by using quantitative methods and have examined the relationships among communication, job satisfaction, and stress [9,10,11]. A person’s subjective perception is influenced by internal factors such as attitudes, behaviors, values, emotions, and opinions. Unlike most aforementioned studies, which predominantly relied on quantitative methods, this study employs Q-methodology, a mixed-methods approach that integrates both quantitative and qualitative research methods. Q-methodology is a research approach to human behavior that involves interpreting individuals’ values and attitudes and recognizing objects from their unique perspectives [12]. We explore subjective perceptions of communication between nurses and surgeons in the OR. In addition, this study can provide basic data for establishing strategies to improve effective communication patterns between nurses and surgeons.

### 1.2. Aim

This study aimed to identify and understand subjective perceptions of communication between nurses and surgeons in the ORs, focusing on their values, beliefs, and attitudes.

## 2. Materials and Methods

### 2.1. Research Design

This study employed a Q-methodology to identify and describe the perceptions of nurse–surgeon communication in the OR.

### 2.2. Q-Methodology

Q-methodology is an integrated research approach that synthesizes the advantages of quantitative and qualitative methods to clarify a subject’s point of view about an attitude, phenomenon, interest, or concern. Subjective viewpoints can be defined by internal factors, such as interpersonal relationships, individual attitudes, impressions, perceptions, feelings, and opinions, rather than external factors [12]. A study using Q-methodology involves the development of the Concourse by using diverse sources, the production of a set of statements known as the Q-sample (or Q-set), the selection of a sample of participants called the P-sample (P-set), and a process of Q-sorting using a bipolar Q-sort table designed as a grid (or data collection table). After these processes, the collected data are analyzed by using varimax rotation for factor analysis. Several factors are eventually identified and labeled by a team of domain experts. The participants are asked to accumulate more information about the two Q statements with which they most agree and disagree, to help interpret the emerging factors. The results of a Q-study not only suggest how people might be approached; they can also predict the success of the approach. To facilitate this analysis, the Q-study protocol was organized to include five sequential steps: (1) the construction of the Concourse, (2) the selection of participants (P-sample) (3) facilitating the Q-sort procedure, (4) Q-factor analysis, and (5) the interpretation of the data from the Q-sorts [13].

### 2.3. Research Procedure

**Figure 1:**
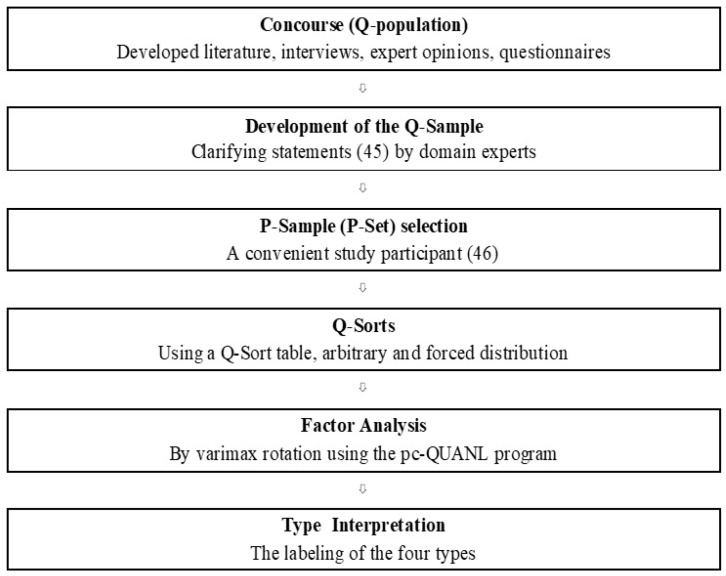
Practical steps of Q-methodology.

The practical steps of the Q-methodology are shown in Figure 1.

#### 2.3.1. Construction of Concourse (Q-Population)

The first box in <Figure 1> displays the Concourse (Q-population) development steps. The Concourse is a collection of knowledge and experience representing the range of views or opinions about the research issue or concept; it becomes the pool of opinions from which the Q-sort statements are drawn [13]. The Concourse (Q-population) consisted of statements that were generated as follows: Previously published studies on nurse–surgeon (physician) communication [1,3,4,9,14,15] were reviewed. Next, in-depth interviews were conducted to extract self-referent statements from three nurses and three surgeons working in the operating room who were not participants in the study. The statements covered broad areas, such as feelings, experiences, and impressions about nurse–surgeon communication. These processes extracted 162 statements as an initial Concourse (Q-population). After reviewing overlapping and ambiguous statements, 87 of the 162 statements were generated as the second Concourse (Q-population). Domain experts, including a Q-methodologist, three researchers, three surgeons (two surgical residents and a professor), and three clinical nurse specialists working in the OR, implemented the procedures for developing the Concourse (Q-population).

#### 2.3.2. Development of a Q-Sample

The 87 statements were reviewed by a panel consisting of three researchers, three nursing professors with expertise in adult and psychiatric nursing, and a methodologist. Then, they were classified according to meaning and theme. A list of 65 statements was edited and reduced to 48 to eliminate the duplication of repetitive viewpoints. After pilot testing, five participants read the statements to evaluate their appropriateness, and three statements were excluded. This step ensured the expression of contrasting viewpoints, involved the rewording and rewriting of statements, and further eliminated superfluous statements. Through this process, a final panel of 45 Q-samples was identified as the most representative and distinctive, and these were chosen for use in the Q-sorting process.

#### 2.3.3. Selection of Study Participants (P-Sample)

In the Q-methodology, 40–60 participants are a reasonable number for sorting, although far fewer might be needed for some studies [12]. For this study, a convenience sample of 46 participants working in the operating room at a tertiary university hospital in Seoul, Korea, was recruited, and they agreed to participate. Participants’ characteristics are shown in Table 1.

#### 2.3.4. Q-Sort by Participants (Q-Sorting)

**Figure 2:**
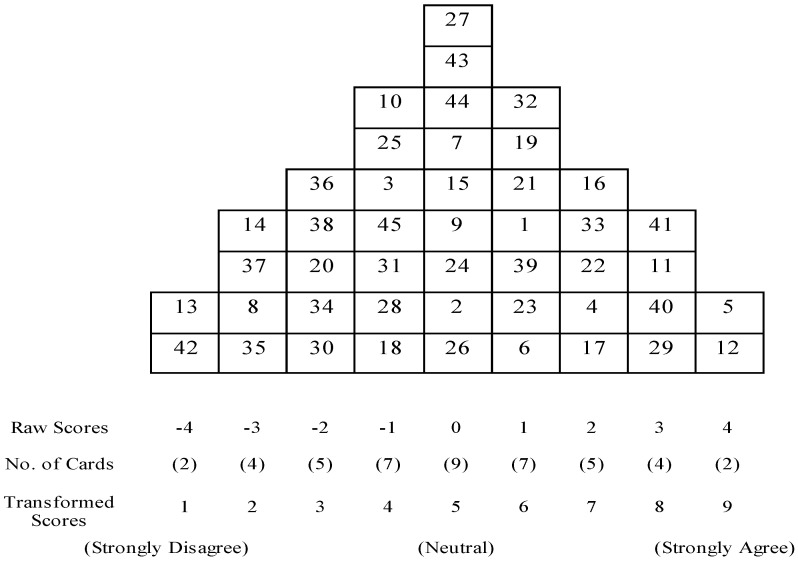
An example of a completed Q-sort table utilized for ranking the Q-sample during the sorting process. Participants allocated one unique statement to each box according to their level of agreement, corresponding to values ranging from −4 to +4 used in the analysis.

In the first step, participants were asked to read the 45 statements selected as Q-samples to determine whether there were questions or items they could not understand. For the Q-sorting process, following the general principle of sorting, the statements they agreed with among the 45 statements were sorted on the right, those they disagreed with on the left, and neutral statements in the middle. Next, of the statements they agreed with, those they most strongly agreed with were placed at +4 and the rest sorted toward the middle. Similarly, of the statements they disagreed with, those they most strongly disagreed with were placed at −4 to surround the neutral statements. Participants wrote why they selected the statements placed at each end (+4, −4) or underwent a post-sorting interview. Participants were also requested to complete a brief survey, including socio-demographic data. The final Q-sort was a matrix representing the participants’ operant subjectivity on the issue under consideration (Figure 2).

### 2.4. Data Collection and Analysis

The researcher collected data between May and June 2023. The Q-sort data were analyzed by using the pc-QUANL program to reveal patterns or groupings after each participant’s scores were entered into the database. Three points are relevant to the analysis of Q-sorts: (1) obtaining eigenvalues of at least 1.0 for the final interpretation, (2) conducting varimax rotation to maximize the variance in each factor, and (3) adopting z-scores as a measure of the standard deviation (statements with a z-score above +1.0 are considered positive views, and below −1.0, negative views). The best estimate for each factor was calculated by using factor weightings demonstrating the extent of an individual Q-sort in each factor. In addition, factor weights that represent the degree of individual Q-sort within each factor were used to calculate the best estimates for each factor. A factor array, participants’ post Q-sorting qualitative comments about the extreme positions for each grid (+4, −4), and demographic data were also used to interpret the factors. The labeling of the factors was carried out by researchers or domain experts [13].

### 2.5. Ethical Considerations

The study was approved by the institutional review board at a university hospital (IRB No. KUH1280047) and was conducted according to the ethical principles of the Declaration. Before this study, the participants were informed of the purpose of this research. Written informed consent was obtained after the participants agreed to participate in this study. The participants were assured of their right of refusal to participate or to withdraw from the study at any stage. All data were separated from identifying characteristics before analysis to achieve confidentiality and anonymity.

## 3. Results

### 3.1. Participants’ Socio-Demographic Characteristics

This study had 46 participants: 35 nurses and 11 surgeons. Their ages ranged from 24 to 58. Among them, 29 individuals had a religious affiliation, and 19 were married (Table 1).

### 3.2. Formation of Q-Types

As shown in Table 2, there were four significant perceptions of nurse–surgeon communication: professional communication, cynical conflict, passive task-oriented communication, and relationship-oriented endurance. The four types accounted for 58.7% of the total variance, including type 1 (38.6%), type 2 (9.9%), type 3 (5.4%), and type 4 (4.8%). The eigenvalues were 15.8, 4.1, 2.2, and 1.9, respectively.

Type 1: Professional communication

Twenty of the forty-six participants were identified as type 1. Type 1 participants reported that effective horizontal communication between all senior and junior members based on mutual trust and a sense of professional duty is important; hence, this type was named ‘professional communication’. The type 1 participant strongly agreed with the following quotations: ‘Q5. Using supportive body language such as a sincere tone, positive attitude, and confidence forms the basis of effective communication’. (Z = 1.79); ‘Q12. Trust is the most important factor in effective communication between individuals’. (Z = 1.78); and ‘Q23. Work becomes enjoyable when surgical progress is swift and seamless due to good teamwork’. (Z = 1.53). On the other hand, these participants strongly disagreed with the following statements: ‘Q43. Unlike in a conversation with my boss, I am not focused when talking with colleagues or juniors’ (Z = −2.08); ‘Q39. I feel like I’m the only one who works hard in the operating room’ (Z = −1.67); and ‘Q40. Verbal abuse brings on nausea, headache, dizziness, and other symptoms’. (Z = −1.42).

Participants who were identified as this type provided illustrative comments about why they placed the statements at the ends of the sorting grid. Examples include the following:

*“Approaching patients with a sense of duty based on mutual trust is the basic requirement for medical professionals.”*

*“If a relationship with colleagues or juniors is difficult, it is because of a lack of inter-communication. Colleagues are those who help through difficult times and it is important to become a senior who approaches the juniors first. Important issues or ideas could be brought up by them.”*

*“When participating in a highly trusted surgery team, mistakes are often handled with patience. The same situation is dealt with differently depending on trust built. Greater confidence and patience are observed when trust is strong.”*

Type 2: Cynical conflict

Type 2, ‘cynical conflict’ reflected how effective communication is possible in the OR only with good communication skills. Specifically, participants who were identified as this type lacked openness and had difficulties communicating with supervisors. They often perceived the nurse–surgeon relationship to be a vertical one, and most had been hurt by verbal abuse in the past. Type 2 participants agreed most strongly with the following statements: ‘Q5. Using supportive body language such as sincere tone, positive attitude, and confidence forms the basis of effective communication’ (Z = 2.18); ‘Q23. Work becomes enjoyable when surgical progress is swift and seamless due to good teamwork’. (Z = 1.85); ‘Q29. I feel a sense of pride when the surgeon trusts me and approaches me for help with equipment and operational procedures’ (Z = 1.69); and ‘Q8. Speaking skills such as a reasonably loud voice and clear and simple speech are important for members to obtain information’. (Z = 1.61). Type 2 showed the strongest disagreement with the following statements: ‘Q43. Unlike in a conversation with my boss, I am not focused when talking with colleagues or juniors’. (Z = −2.35) and ‘Q13. I believe that the use of smartphones and messengers interferes with personal communication’. (Z = −1.51).

Participants who identified as this type provided illustrative comments about why they placed the statements at the ends of the sorting grid. Examples include the following:

*“Whether I am working with seniors or juniors when an emergency arises, I unknowingly become irritated and demanding. I think others would act the same way.”*

*“Urgent situations arise often, and, considering the time required to move about in a wide space, situations often call for just hearing the solution and quickly moving on. Also, accurate communication is important since it is easy to misunderstand what is being said with the loud noises from surrounding machines or being highly focused on the surgery.”*

Type 3: Passive task-oriented communication

Type 3, ‘passive task-oriented communication’ reflected how communication based on trust was carried out with participants’ colleagues, juniors, and supervisors. They considered the upbeat atmosphere between team members important. Still, they failed to recognize their own independence or autonomy and could not engage in the smooth mutual exchange of ideas or active conversation. Type 3 participants strongly agreed with the following statements: ‘Q12. Trust is the most important factor in effective communication between individuals’ (Z = 2.60) and ‘Q23. Work becomes enjoyable when surgical progress is swift and seamless due to good teamwork’. (Z = 2.08). However, they strongly disagreed with the following statements: ‘Q43. Unlike in a conversation with my boss, I am not focused when talking with colleagues or juniors’ (Z = −2.12) and ‘Q6. It is effective to offer the solution first and then explain the situation later in the OR’ (Z = −1.67).

Participants who were identified as this type provided the following illustrative comments about why they placed the statements at the ends of the sorting grid, as follows:

*“There might be a bad atmosphere because everyone is performing a difficult surgery together, but we can probably laugh more since we share the difficulty. I believe a group based on trust is more positive.”*

*“Due to the unresolved vertical relationship between nurses and surgeons, which persists even among nurses, I tend to go along with others rather than trying to resolve differing opinions.”*

*“Stating solutions initially can create misunderstandings and explaining things that happen after a solution has been reached can create confusion between each other. That’s why I believe step-by-step conversation is fundamental.”*

Type 4: Relationship-oriented endurance

Type 4 was designated as ‘relationship-oriented endurance’. Participants of this type emphasized non-verbal communication such as intonation, rhythm, facial expressions, and gestures. They possessed individualistic ambivalence and tried to avoid mutual interactions with their supervisors, but at the same time, they considered their relationship with their bosses important. The statements that type 4 participants agreed most strongly with were ‘Q14. While communication heavily relies on words and language, non-verbal cues such as intonations, expressions, and gestures are even more important’. (Z = 2.12); ‘Q18. In the operating room, if I am not directly related to a matter, I am not concerned about it’. (Z = 2.01); and ‘Q42. I usually suppress my anger when my bosses or subordinates speak rudely to me’. (Z = 1.81). Meanwhile, type 4 participants strongly disagreed with the following statements: ‘Q44. In many cases, I did not know what most of the surgical team knew’. (Z = −1.94) and ‘Q1. I have difficulty speaking/thinking using language and gestures to communicate with the others present’. (Z = −1.78).

Participants who were identified as this type provided illustrative comments about why they placed the statements at the ends of the sorting grid, as follows:

*“Controlling the impact of one’s opinion through verbal intonation, rhythm, facial expressions, and gestures when praising or criticizing during mutual communication can allow much more flexible communication.”*

*“I usually refrain from actively expressing my emotions when bosses or junior staff say something rude since it may lead to relationship problems at the workplace.”*

Consensus views regarding communication among the four types

The four types showed consensus despite having their own independent characteristics. All types of statements that showed the highest agreement were ‘Q29. I feel a sense of pride when the surgeon trusts me and approaches me for help with equipment and operational procedures’. (Z = 1.37). The statements that showed the highest disagreement were ‘Q13. I believe that the use of smartphones and messengers interferes with personal communication’. (Z = −1.47) and ‘Q1. I have difficulty speaking/thinking using language and gestures to communicate with the others present’. (Z = −1.31).

Overall, nurses and surgeons in the OR took pride in their active communication during procedures and in sharing important information based on mutual trust. They recognized that various methods, including smartphones and messaging applications, can achieve effective communication.

## 4. Discussion

Effective communication in the operating room (OR) significantly influences the OR team’s performance, which in turn affects patient safety and treatment outcomes [6]. Until recently, most healthcare institutions and previous studies have employed situation, background, assessment, and recommendation (SBAR) as a communication tool for healthcare professionals in clinical and medical education settings [14,15]. This study utilized the Q-methodology, which differs from the quantitative studies previously mentioned. The Q-methodology concentrates on human beliefs, values, and emotions. The aim was to identify subjective perceptions of communication between nurses and surgeons and to understand the various characteristics of communication types in the OR. This approach provides baseline data that can be used to develop effective communication programs in clinical practices. Type 1 (professional communication) had the most participants (n = 20) among all types and referred to how participants engaged in horizontal communication with a sense of responsibility and openness based on trust. Horizontal communication possesses the elements of mutuality and professionalism and is mostly observed in highly specialized organizations; furthermore, it is a form of communication for exchanging necessary information to reduce unnecessary conflict and encourage cooperation [16,17]. Type 1 is an ideal communication pattern that satisfies most of the factors associated with effective nurse–surgeon communication. Type 1 participants agreed with the following statement to a greater degree than did participants of the other types: ‘Q35. There are many opportunities to discuss patient care problems by sharing case details with colleagues’. Furthermore, they most often disagreed with statement ‘Q25. I work patiently and often ignore the other surgical team members’ impatience and rudeness’., which is an issue related to the openness of communication. The openness of nurse–surgeon/nurse–nurse communication and mutual understanding between occupations have shown strong correlations with communication satisfaction [18,19].

Moreover, openness in communication prevents silence that interferes with patient safety and clarifies previously unclear situations through mutual understanding between professionals, all of which have a major impact on satisfaction with communication [6,7]. Therefore, openness in communication should be highlighted during the development process for communication education programs. Type 1 mostly comprised nursing managers with considerable experience (between 7 and 17 years). This accords with previous research [16], which indicated that those with the highest degree of nurse–surgeon collaboration were 35 years or older, were Head Nurses, and had more than 10 years of clinical experience at their current hospital. It also aligns with a study [20] which reported that nurses over 30, those with diplomas, those with more than 10 years of experience, and those in supervisory positions had more positive perceptions of the quality of nurse–physician communication. In summary, participants of type 1 engaged in professional communication with openness and a high degree of collaboration. Thus, they can be considered role models, although a thorough analysis and subsequent application of type 1 communication characteristics are required to obtain more evidence for the development of standardized nurse–surgeon communication programs.

Participants identified as type 2 (cynical conflict) believed that skills in conciseness and clarity are essential to effective communication in the OR. However, these individuals often lack openness, struggle to communicate with their superiors, view the nurse–surgeon relationship as hierarchical, and have experienced verbal abuse in the past.

In contrast, Cheong [21] reported that nurses generally feel satisfied with vertical communication with their superiors but less so regarding the organizational climate. This satisfaction with vertical communication could indicate positive changes in today’s hospitals concerning work-related information and guidelines, along with greater open-mindedness among healthcare staff. This marks a shift away from the downward communication style commonly seen in the bureaucratic and static organizations of the past [22]. The Q-statements that type 2 participants most strongly agreed with included ‘Q40. Verbal abuse brings on nausea, headache, dizziness, and other symptoms’. and ‘Q39. I feel like I’m the only one who works hard in the operating room’.

Chang et al. [8] found that the level of verbal abuse from surgeons and superiors is particularly high in the OR, and this abuse from superiors is significantly associated with leaving the job, which aligns with the characteristics of type 2 participants. As a result, staff in the OR are more susceptible to verbal abuse and stress compared with those in other units. Promoting effective communication within the surgical healthcare team, organizational support and efforts are crucial [3]. To enhance communication for type 2 participants, it is essential to cultivate a team atmosphere of respect and mutual concern, both at the individual and organizational levels.

Type 3 (passive task-oriented communication) participants believed that effective communication should be built on trust with supervisors, colleagues, and subordinates, and they also valued a positive team atmosphere. However, they often failed to recognize their independence or autonomy and could not engage in the smooth mutual exchange of ideas or active conversation. In other words, type 3 participants had high openness to communication but engaged in such communication with restricted independence and autonomy; hence, they were assumed to have fundamentally similar communication attitudes to type 2 participants. A previous study [22] noting that health professionals generally have strong intentions and recognition of patient safety when participating in decision making serves as evidence for the importance of autonomy [23], which is lacking in participants of type 3. As the collaboration among healthcare organization members significantly impacts both the members themselves and the patients in their care, fostering teamwork is crucial. However, collaboration and communication between nurses and physicians remain weak [7]. A previous study [23] has reported that nurse–physician collaboration was highly correlated with job autonomy (r = 0.242); thus, establishing measures to increase the collaborative relationship between nurses and surgeons is of utmost importance in increasing job autonomy and supports the need for interventions aimed at enhancing nurse–surgeon collaboration in type 3 participants.

Type 4 (relationship-oriented endurance) emphasizes non-verbal communication such as intonation, rhythm, facial expressions, and gestures. Type 4 tended to avoid mutual interactions with their bosses but, at the same time, often considered their relationship with their bosses important. A study by Cheong [21] found that nurses generally exhibited low competency in self-expression, including assertion, expressiveness, interaction management, and reasoning. This aligns with the characteristics of type 4 participants, who were generally described as individualistic, passive, and close-minded. Therefore, it is necessary to develop systematic self-assertion training programs to foster active and positive communication competencies among nurses. Previously published studies [7,17] noted that among leadership actions taken by surgeons, the highest frequencies were observed in guidance and support followed by communication and collaboration. The findings indicate that a leader must establish an environment that fosters effective communication. Additionally, the findings suggest that leaders play a particularly significant role for individuals similar to those in type 4, who highly value their relationships with their superiors.

Meanwhile, the statement that OR nurses and surgeons both agreed with was ‘Q29.I feel a sense of pride when the surgeon trusts me and approaches me for help with equipment and operations’; this suggests a positive pattern of information exchange and communication in their professional fields. Such an attitude was consistent with previous studies [3,7,24] that indicated that mutual trust between nurses and physicians significantly influences the degree of satisfaction with nurse–physician collaboration. On the other hand, they strongly disagreed with statement ‘Q13. I believe that the use of smartphones and messengers interferes with personal communication’. This finding highlights the significance of communication media, aligning with previous research indicating that wireless devices and smartphones improve communication accuracy and satisfaction while reducing wasted time and external disturbances [25]. OR nurses reported improved communication among healthcare team members using their devices to communicate patient information via text messaging, calling, and picture and video functions.

Communication occurs frequently in very complex patterns in hospital ORs. Compared with other departments, nurses working in the OR have diminished job autonomy, poor recognition of patient safety, and high turnover intention [26] and often receive high levels of verbal abuse by surgeons and supervisors, making them vulnerable to stress [27]. Improving these OR-related issues can benefit patients, healthcare professionals, and organizations through professional and effective communication. As our findings indicate, developing a communication manual to consider the subjectivity of OR nurses and surgeons is necessary. Additionally, there is a need to actively explore communication enhancement strategies that leverage various information media, such as social networking services like Twitter, Facebook, and Instagram. This is supported by the consensus views on the types of communication identified in our study.

Consequently, it is crucial to establish efforts and support systems at both organizational and individual levels to improve effective communication skills through practical enhancement programs.

### 4.1. Strengths and Limitations

The strength of this study lies in its identification of unique perceptions regarding nurse–surgeon communication in the OR. This study provides practice-based evidence that clarifies the competencies required for effective communication in human resource management within clinical settings. The findings suggest hospital and nursing managers with useful data to promote understanding and develop effective educational programs on communication. Some limitations of the study should be considered. First, we used convenience sampling, which means that ours was not a representative population sample; thus, the results of this study cannot be extrapolated to the general population. Second, a potential limitation is that the findings should be applied cautiously in other cases because the study participants were Koreans living in Asia. Despite these limitations, this study contributes valuable insights into the perceptions of nurse–surgeon communication in the sociocultural context of Korea.

### 4.2. Implications for Clinical Practice and Research

Our findings have several practical implications. This study suggests that a differentiated approach is needed to plan and perform interventions to improve communication skills among healthcare professionals. Hospital and nursing managers need to assess the communication skills of healthcare professionals to understand their communication styles, thereby increasing job satisfaction and reducing turnover rates.

The findings highlight the importance of developing customized interventions that consider the subjective characteristics of each type. This study provides baseline data for improving communication skills by identifying communication patterns between nurses and surgeons in the OR. Moreover, these findings offer valuable data to guide the development of educational programs, protocols, and policies. Additionally, this study informs healthcare organizations in other countries regarding the communication traits of Korean healthcare professionals.

## 5. Conclusions

The most important finding of this study is that it identified health professionals’ subjective perceptions and the characteristics of each type of communication in the OR. This study provides essential data for developing effective communication skills education programs by identifying perceptions of communication between nurses and surgeons and analyzing their structural characteristics while working in ORs. Developing communication skills education programs that consider the unique subjectivities of nurses and surgeons, based on the four types discovered in the present study, would be beneficial for health professionals working in extremely sensitive and complex environments.

This study provides further evidence highlighting the necessity of fostering organizational cultures within hospitals and operating rooms. Such cultures play a critical role in delivering high-quality care that ensures patient safety, ultimately reducing turnover rates and enhancing job satisfaction among healthcare professionals [20]. Hospital and nursing managers are encouraged to apply the findings of this study to improve human resource management practices and to promote the safety and quality of patient care.

### Future Research

Based on the findings of this study, we suggest several directions for future research aimed at improving communication between nurses and surgeons. First, it is essential to examine communication perceptions across various hospital units, including outpatient departments (OPDs) and intensive care units (ICUs), to gain a comprehensive understanding of communication dynamics in different clinical settings. Second, future research should focus on developing tailored continuing education programs that account for different communication styles in ORs, particularly in the context of nursing personnel management. Furthermore, studies should assess the effectiveness of these programs in enhancing interdisciplinary collaboration. Third, expanding the participant pool and conducting international comparative studies on communication patterns between nurses and surgeons would provide valuable insights into cross-cultural differences and best practices.

These investigations would contribute to optimizing hospital services, thereby enhancing both patient safety and the satisfaction of healthcare professionals.

## Figures and Tables

**Table 1:** Participants’ socio-demographic characteristics, factor weights, eigenvalue, variance, and cumulative percentage (N = 46).

Type (*n*)	P-No.	Factor Weight	Age (yr)	Gender	Education	Religion	Marital Status	Job	Position	Career (yr)
	32	1.8638	40	M	PhD	None	Married	Surg	Professor	12
	25	1.7928	25	F	Bachelor	Protestant	Unmarried	RN	RN	2
	10	1.6788	39	F	Bachelor	Buddhism	Unmarried	RN	CN	16
	35	1.6477	35	M	Master	Protestant	Married	Surg	Fellow	6
	9	1.5870	36	F	Bachelor	Buddhism	Unmarried	RN	CN	15
1 (n = 20)	4	1.4022	38	F	Bachelor	None	Married	RN	CN	15
	1	1.3849	38	F	Master	Protestant	Married	RN	HN	17
	36	1.3539	37	M	PhD	Buddhism	Unmarried	Surg	Fellow	6
	7	1.3462	35	F	Bachelor	Protestant	Unmarried	RN	CN	13
	30	1.3374	34	F	Bachelor	Protestant	Married	RN	CN	12
	8	1.3252	28	M	Bachelor	None	Unmarried	RN	RN	2
	14	1.2218	33	F	Bachelor	Protestant	Unmarried	RN	RN	7
	40	0.9966	28	M	Bachelor	Catholic	Unmarried	Surg	Internist	1
	39	0.8530	38	M	Master	Catholic	Married	Surg	Professor	9
	27	0.8404	27	F	Bachelor	Protestant	Unmarried	RN	RN	3
	11	0.8241	36	F	Master	Catholic	Married	RN	CN	11
	18	0.8011	40	F	Bachelor	None	Married	RN	RN	18
	5	0.6268	27	M	Bachelor	Protestant	Unmarried	RN	RN	3
	43	0.5247	38	F	Master	None	Married	RN	CN	13
	46	0.5032	36	f	PhD	Catholic	Married	Surg	Resident	6
2 (n = 15)	2	1.4157	30	F	Master	None	Unmarried	RN	RN	8
	3	1.3537	29	F	Bachelor	Protestant	Unmarried	RN	RN	4
	6	1.2454	30	F	Bachelor	None	Unmarried	RN	RN	7
	12	1.0252	31	F	Bachelor	Catholic	Unmarried	RN	RN	7
	21	0.9477	28	F	Bachelor	None	Unmarried	RN	RN	2
	15	0.9439	33	F	Bachelor	None	Unmarried	RN	RN	9
	17	0.9072	29	F	Bachelor	None	Unmarried	RN	RN	8
	41	0.8445	32	F	Bachelor	Protestant	Unmarried	Surg	Resident	2
	16	0.8425	30	F	Bachelor	Protestant	Married	RN	RN	6
	22	0.7411	33	M	Bachelor	Protestant	Married	RN	RN	6
	29	0.7374	30	F	Bachelor	None	Unmarried	RN	RN	7
	26	0.6825	40	F	Bachelor	None	Married	RN	CN	17
	23	0.6519	30	F	master	Protestant	Married	RN	RN	10
	37	0.3111	58	M	PhD	Protestant	Married	Surg	Head	32
	44	0.2914	35	M	PhD	None	Unmarried	Surg	Resident	5
	31	1.3783	27	F	Bachelor	Protestant	Unmarried	RN	RN	3
3 (n = 7)	24	1.0469	26	F	Bachelor	Catholic	Unmarried	RN	RN	3
	34	1.0440	35	M	Master	None	Married	RN	Resident	4
	38	0.7960	33	F	Master	Catholic	Unmarried	RN	Resident	3
	19	0.7614	26	M	Bachelor	Protestant	Unmarried	RN	RN	1
	33	0.7338	36	M	PhD	None	Married	Surg	Resident	4
	45	0.7217	38	M	PhD	None	Unmarried	Surg	Fellow	6
4 (n = 4)	28	2.5630	31	M	Bachelor	Protestant	Married	RN	RN	6
	13	2.4274	31	M	Bachelor	Protestant	Married	RN	RN	5
	20	0.6916	30	F	Bachelor	Protestant	Unmarried	RN	RN	4
	42	0.6712	27	F	Bachelor	None	Unmarried	RN	RN	3
			**Type 1**		**Type 2**		**Type 3**		**Type 4**	
Eigenvalue			15.8261		4.1229		2.2132		1.9205	
Variance (%)			0.3867		0.0984		0.0544		0.0476	
Cumulative (%)			0.3867		0.4851		0.5395		0.5871	

Note: P-No. = participant number; RN = Registered Nurse; CN = Charge Nurse; HN = Head Nurse; Dept. = department; yr = year; Surg = surgeon.

**Table 2:** Q-statements on communication in the OR and Z-scores for each type (N = 46).

Q-Statement	Z-Score			
	Type 1 (n = 20)	Type 2 (n = 15)	Type 3 (n = 7)	Type 4 (n = 4)
Q1. I have difficulty speaking/thinking using language and gestures to communicate with the others present. †	**−1.25**	**−1.32**	−0.88	**−1.78**
Q2. Communication among members in our operating room is sufficiently clear for us to achieve our common goal.	0.39	−0.85	0.52	−0.38
Q3. There is a lack of clear communication when we have a new task or a difficult situation, or if our opinions differ.	−0.52	0.27	−0.21	−0.26
Q4. When I share information and make decisions, I actively communicate to resolve any differences in our opinions.	0.26	0.71	**−1.18**	−0.06
Q5. Using supportive body language such as a sincere tone, positive attitude, and confidence forms the basis of effective communication.	**1.79**	**2.18**	**1.11**	0.46
Q6. It is effective to offer the solution first and then explain the situation later in the OR.	−0.63	−0.29	**−1.67**	0.37
Q7. The mood in our operating room is pleasant due to effective communication.	0.17	−0.43	0.19	−0.43
Q8. Speaking skills such as a reasonably loud voice and clear and simple speech are important for members to obtain information.	0.86	**1.61**	0.64	0.11
Q9. Our operating room is good at brief and accurate communication and providing prompt feedback.	0.28	−0.23	0.25	0.17
Q10. Our operating room’s medical team communicates all surgery-related information in a relatively relaxed ambiance.	0.37	−0.84	−0.18	−0.57
Q11. To effectively communicate with the medical team, job performance is more important than an individual’s qualities.	−0.94	0.43	0.94	−0.06
Q12. Trust is the most important factor in effective communication between individuals.	**1.78**	**1.56**	**2.60**	**1.20**
Q13. I believe that the use of smartphones and messengers interferes with personal communication. †	**−1.15**	**−1.51**	**−1.50**	**−1.73**
Q14. While communication heavily relies on words and language, non-verbal cues such as intonations, expressions, and gestures are even more important.	0.50	0.21	0.82	**2.12**
Q15. The individual’s focus on the mission and a positive frame of mind are necessary for effective communication with the medical team.	**1.35**	**1.52**	**1.89**	−0.02
Q16. Our operating room is confident about standardization, the division of roles, and homogenized terminology.	0.41	−0.63	0.06	0.60
Q17. Our operating room is a comfortable and adequate space for communication between team members.	0.44	−0.65	0.43	0.17
Q18. In the operating room, if I am not directly related to a matter, I am not concerned about it.	−0.95	−0.58	−0.54	**2.01**
Q19. I often perceive a superior–subordinate dynamic between the surgeons and nurses in the operating room.	−0.82	0.62	−0.05	−0.45
Q20. I discuss my plans with the surgical team, and they respect my opinions.	**1.14**	0.32	0.19	0.07
Q21. As a surgical team, we share a good rapport; this helps us conduct operations smoothly.	**1.35**	0.03	0.67	0.65
Q22. Even if surgery is difficult, the surgical team members care about one another, and the atmosphere is friendly.	0.92	−0.47	**1.33**	**−1.25**
Q23. Work becomes enjoyable when surgical progress is swift and seamless due to good teamwork.	**1.53**	**1.85**	**2.08**	−0.19
Q24. My surgical team supports me completely and comes up with solutions in unexpected situations; they are not impatient and let me work at my own pace.	−0.62	−0.60	**−1.20**	**−1.37**
Q25. I work patiently and often ignore the other surgical team members’ impatience and rudeness.	**−1.39**	0.59	0.57	0.77
Q26. We share our operating room’s work difficulties with seniors and juniors; this is a pleasant practice.	0.12	−0.51	0.34	0.24
Q27. The operating room team addressed our mistakes privately instead of in front of others.	−0.09	−0.69	**−1.00**	**−1.30**
Q28. When my boss understands and recognizes my difficulties, I am more motivated and enjoy my work.	0.36	−0.80	−0.54	**1.24**
Q29. I feel a sense of pride when the surgeon trusts me and approaches me for help with equipment and operational procedures. †	**1.00**	**1.69**	**1.77**	**1.01**
Q30. Our operating room believes that the nurses’ training period is sufficient.	−0.51	**−1.39**	−0.30	−0.63
Q31. I feel that work allocation is inappropriate, especially when working with seniors.	−0.88	0.35	0.30	−0.72
Q32. Regulations reflect the opinions of the operating room nurses, and there are many opportunities to participate in decision making.	0.02	−0.97	−0.58	0.70
Q33. My boss understands our problems and tries to solve them.	0.28	−0.95	−0.16	0.75
Q34. Our teamwork and cooperation among operating room nurses are good.	0.89	−0.06	0.28	0.31
Q35. There are many opportunities to discuss patient care problems by sharing case details with colleagues.	0.07	−0.78	**−1.22**	−0.88
Q36. It’s beneficial to converse with colleagues I have worked with.	0.88	0.91	0.20	0.77
Q37. At work, I have been given relative independence and autonomy.	0.73	0.47	**−1.23**	0.65
Q38. My work capabilities affect many people.	**1.36**	**1.42**	−0.64	0.31
Q39. I feel like I’m the only one who works hard in the operating room.	**−1.67**	0.05	−0.77	**−1.25**
Q40. Verbal abuse brings on nausea, headache, dizziness, and other symptoms.	**−1.42**	0.65	−0.69	**−1.44**
Q41. When I talk to someone, I tend to evaluate their words based on non-verbal indicators (appearance and behavior, intonation, etc.).	**−1.36**	**−1.20**	−0.06	0.08
Q42. I usually suppress my anger when my bosses or subordinates speak rudely to me.	**−1.00**	0.66	0.30	**1.81**
Q43. Unlike in a conversation with my boss, I am not focused when I talk with colleagues or juniors.	**−2.08**	**−2.35**	**−2.12**	**−1.30**
Q44. In many cases, I did not know what most of the surgical team knew.	**−1.34**	−0.61	−0.84	**−1.94**
Q45. We typically do not challenge our boss’s opinions in our operating room.	−0.61	0.61	0.03	**1.42**

Note: † = consensus statement; boldface = factor z-scores above +1.0 or below −1.0; OR = operating room.

## Data Availability

The data presented in this study are available upon request from the first and corresponding author. Due to ethical issues, they are not publicly available.
